# Inhalable Thioflavin S for the Detection of Amyloid Beta Deposits in the Retina

**DOI:** 10.3390/molecules26040835

**Published:** 2021-02-05

**Authors:** Shawn M. Barton, Eleanor To, Baxter P. Rogers, Clayton Whitmore, Manjosh Uppal, Joanne A. Matsubara, Wellington Pham

**Affiliations:** 1 Vanderbilt University Institute of Imaging Science, Vanderbilt University Medical Center, Nashville, TN 37232, USA; shawn.m.barton@vanderbilt.edu (S.M.B.); baxter.rogers@vanderbilt.edu (B.P.R.); clayton.whitmore@vumc.org (C.W.); 2 Department of Radiology and Radiological Sciences, Vanderbilt University Medical Center, Nashville, TN 37232, USA; 3 Department of Ophthalmology and Visual Sciences, The University of British Columbia, Eye Care Center, Vancouver, BC V5Z 3N9, Canada; ect@mail.ubc.ca (E.T.); manjosh@mail.ubc.ca (M.U.); 4 Department of Biomedical Engineering, Vanderbilt University, Nashville, TN 37235, USA; 5 Department of Psychiatry and Behavioral Sciences, Vanderbilt University Medical Center, Nashville, TN 37212, USA; 6 Vanderbilt Brain Institute, Vanderbilt University, Nashville, TN 37232, USA; 7 Vanderbilt Ingram Cancer Center, Nashville, TN 37232, USA; 8 Vanderbilt Institute of Chemical Biology, Vanderbilt University, Nashville, TN 37232, USA; 9 Vanderbilt Institute of Nanoscale Science and Engineering, Vanderbilt University, Nashville, TN 37235, USA; 10 Vanderbilt Memory and Alzheimer’s Center, Vanderbilt University Medical Center, Nashville, TN 37212, USA

**Keywords:** Alzheimer, molecular imaging, retinal imaging, probes

## Abstract

We present an integrated delivery technology herein employing the aerosolized method to repurpose thioflavin S for imaging amyloid beta (Abeta) deposits in the retina as a surrogate of Abeta in the brain for early detection of Alzheimer’s disease. The data showed that wild type (WT) mice also have Abeta deposits in the retinae, albeit much less than 5XFAD mice. Further, only in 5XFAD mice, significant Abeta deposits were found associated with retinal ganglion cells (RGCs) in whole-mount and cross-section data. Furthermore, the fluorescent signal depicted from thioflavin S corroborates with Abeta immunohistochemistry staining information. Overall, this probe delivery via inhalation method is also applicable to other Abeta-binding molecules, such as Congo red, curcumin, and thioflavin T. The advantage of imaging retinal amyloid deposits compared to the brain counterparts is that the eye is easily accessible by in vivo imaging and it reduces the effort to design a probe that must cross the formidable blood-brain barrier.

## 1. Introduction

Alzheimer’s disease (AD) remains among the very few illnesses that have been known for over a century, but remains without a clear and effective method for diagnosis and to date, there is no approved disease-modifying therapeutic. The concerns have been compounded not only by an increase in the incidence of AD due to the aging population but by the repeated failure of clinical trials attempted to find a cure for the disease. The mechanism underlying AD pathogenesis is unknown; however, a large body of literature suggests that the formation of extracellular amyloid beta (Abeta) plaques in the brain is the culprit behind neuronal toxicity and tissue atrophy [1]. Since the formation of Abeta is one of the underlying mechanisms implicated in AD, detection of Abeta pathology, particularly at the early stage, would be a real impetus for the treatment and assessing the response to therapy.

At present, several imaging modalities have been used to detect Abeta plaques in clinical trials, including positron emission tomography (PET), single-photon emission computerized tomography (SPECT), and magnetic resonance imaging (MRI). For PET probes, Pittsburgh compound B ([^11^C]PIB), an analog of thioflavin T, is the most characterized as an in vivo PET radiotracer since it was reported approximately two decades ago [2]. The ^11^C PIB-PET is moderately effective for the diagnosis of mild cognitive impairment due to AD-high likelihood [3]. The probe was also used to assess the efficacy of Bapineuzumab, a humanized monoclonal antibody that binds Abeta plaques in clinical trials [4]. In addition to [^11^C] PIB probe, a variety of other PET probes have recently emerged [5,6].

Radioiodinated and technetium-99 tracers have also been reported as potential SPECT imaging agents for imaging Abeta plaques [7,8,9,10]. Clinical SPECT imaging using 99mTc-HMPAO tracers suggests that the technique may be a useful adjunct to clinical evaluation and a more sensitive biomarker than standard structural imaging [8]. In particular, SPECT imaging is greatly improved by the integrated use of visual assessment and statistical analysis. Non-radioisotopic probes for imaging AD include [^19^F] fluoride Congo red and [^19^F] curcumin mimetics [11,12]; perfluoro agents, such as styrylbenzoxazole analogs have also emerged [12]. Among the tested compounds, the styrylbenzoxazole compound linked with seven ethylene glycol groups in the PEG (polyethylene glycol) chain showed significant [^19^F]MR signals in the brains of AβPPswe/PS1dE9 mice compared to wild type (WT) counterparts. Other techniques using exogenous probes are also being studied, such as manganese chloride for manganese-enhanced MRI (MEMRI). In this approach, following systemic administration of manganese, in vivo detection of layers in different areas of the brain can be performed, including the olfactory bulb, cortex, hippocampus, and cerebellum [13]. MEMRI offers a powerful tool to measure neuronal function [14] in the early stages of neurodegeneration, including AD [15]. Overall, these techniques have demonstrated potential and advantages for imaging Abeta both in preclinical and clinical settings. In particular, PET and MRI have been used similarly to monitor the efficacy of experimental therapeutics on preventing plaque formation and the neurodegenerative changes associated with AD.

Yet, imaging to detect Abeta in the brain has significant drawbacks, including high cost due to the use of advanced imaging modalities. Additionally, probe development is hindered by the blood-brain barrier (BBB), which limits the penetration of many experimental agents, including Thioflavin S (ThioS) [16]. An alternative to these limitations is afforded by the recent discovery of Abeta deposition in the retinae of humans diagnosed with AD [17], as well as in the retinae of preclinical mouse models [18]. In a clinical study, comparing the retinal functions of AD patients (*n* = 20) versus healthy people (*n* = 17) using PET, MRI, and optical coherence tomography (OCT), the work showed that the retinae are also found with increased Abeta load in early AD [19]. The proof-of-concept clinical imaging for the detection of Abeta in the retinae was explored recently using a commercially available curcumin-based Longvida probe [20].

Recently, our lab demonstrated that an aerosolized curcumin analog could be delivered to the brain bypassing the BBB and binding to Abeta in the 5XFAD mice [21,22]. Capitalizing from that experience, we hypothesized that the use of inhalable drugs via aerosolization could also target retinal pathology, and combined with retinal optical imaging, could be used to detect retinal Abeta noninvasively. We present herein a simplified implementation of the aerosolized method to overcome a long-standing technical challenge regarding the in vivo application of Abeta-binding molecules, such as ThioS. This compound has a remarkable binding affinity to Abeta and is used in numerous assays to visualize Abeta for AD research. However, ThioS cannot penetrate the BBB due to the possession of a positive charge moiety. No report so far has described the use of ThioS for the detection of Abeta in vivo. Generating a ThioS aerosolized formulation, we demonstrate herein the compound can bind and report Abeta deposits in a retinae of transgenic mouse model of AD.

## 2. Results

### 2.1. The ThioS Formulation Remains Unchanged after Going through an Atomizer

**Figure 1:**
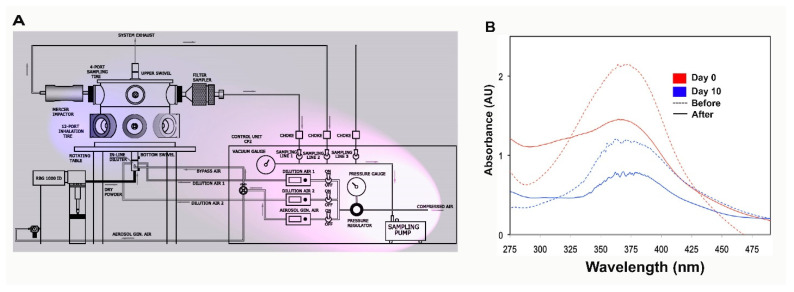
The design of the semi high-throughput aerosolization device for in vivo treatment of small animal models and formulation characterization. (**A**) The control module (pink shade) is comprised of a precision sampling pump, a controlled source of compressed air, which feeds directly to the aerosol generation line or other lines for dilution air when it is necessary to thin the aerosolized solution. The aerosol chamber (blue shade) is equipped with a polyvinyl fluorinated polymer cross-flow atomizer (single-pass atomizer) atomizer on the bottom of the chamber. The generated aerosol will be conveyed through an L-shaped conveyor, which projects directly to a stainless steel inhalation chamber designed to collect the aerosol and distribute it equally to gasket-tight sealed outlets capable of serving 5 animals simultaneously; (**B**) testing the stability of Thioflavin S (ThioS) formulation in dd water at the concentration of 2% (*w*/*v*). The solution was split into two batches; one was immediately tested. The other was kept in the dark at room temperature and tested with an identical procedure 10 days later. The absorbance of ThioS at lambda max 374 nm in water was assessed using a spectrophotometer before and after passing the solution through the atomizer on day 0 (red curves) and day 10 (blue curves). The dash lines indicate the solution before being aerosolized, while continuous lines represent the solution after being aerosolized.

The first task in this work requires optimization of the nebulizer parameters (Figure 1A) and confirmation that a stable formula for aerosol generation is used. A clear solution of 2% ThioS solution in dd water (double distilled water) (*w*/*v*) was prepared. The rationale behind the use of water in the nebulization formula is because of its neutral pH, which helps to maintain substance stability, as well as to prevent increased viscosity. The ThioS aerosol quality was determined by collecting aerosolized products on filter papers for analysis using a spectrophotometer and comparing the absorbance with that of the original ThioS formula that has not gone through the atomizer. The absorbance lambda max (λ_max_) at 374 nm of the collected ThioS aerosol on days 1 and 10 were identical, suggesting that the formulation is stable over storage at an extended period of time at room temperature. Further, based on the absorbance spectra, no substance degradation was found after passing through the atomizer, demonstrating the stability of the aerosol formulation (Figure 1B).

### 2.2. ThioS Detects Retinal Abeta Deposits in 5XFAD Mice

#### 2.2.1. Whole-Mount Analysis

**Figure 2:**
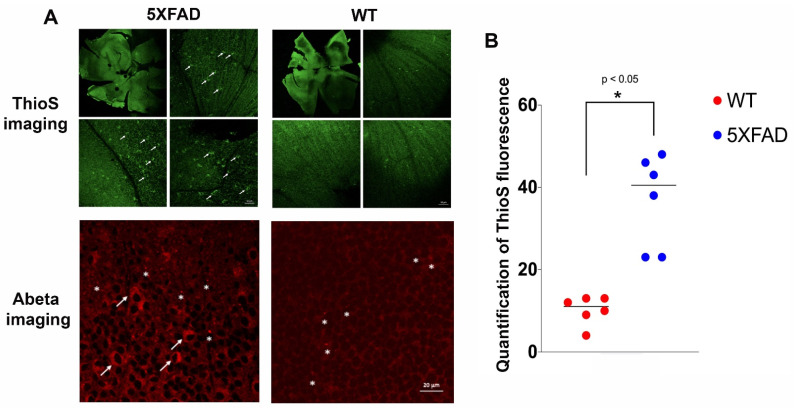
Representative images of confocal fluorescence imaging of retinal whole-mount of treated wild type (WT) versus 5XFAD mice. (**A**) ThioS fluorescence (green, 488 nm channel) are prominent in different areas of the 5XFAD retinae compared to those from WT counterparts. There are some signals in the WT retinae, although much less, but detectable. Selected areas are magnified to show more detailed information. The fluorescence signal appears in the intracellular compartment of the retinal ganglion cells (RGC, arrows). These results corroborate with the retinal amyloid beta (Abeta) immunohistochemistry (red, 546 nm channel) shown in the bottom panels in which RGCs are labeled in 5XFAD (arrows) and extracellular Abeta deposits are also present (asterisks) in both 5XFAD and WT mice; (**B**) quantification of the ThioS signal was performed on retinal sections from WT and 5XFAD mice, and the signal distribution was scored on an ordinal scale after thresholding using Otsu method, and presented in the vertical scatterplot. An asterisk indicates significant differences between WT vs. 5XFAD (*p* < 0.05).

In this proof-of-principle study, we wanted to assess whether 5XFAD mice express Abeta deposits in the eyes. The ocular tissues of aerosolized ThioS-treated 5XFAD (*n* = 3) versus control WT (*n* = 2) mice were collected after cardiac perfusion. The flattened whole-mount retina enables visualization of the topological arrangement of Abeta deposits. It is apparent that Abeta deposits, as depicted by ThioS fluorescence, could be found in both WT and 5XFAD, albeit with a remarkable significance in the latter (Figure 2A, green, 488 nm channel). The fluorescent signal is located in separate areas across the regions of the retinae, including extracellular matrix and in the retinal ganglion cells (RGC). In stark contrast, only a few fluorescent signals assuming Abeta deposits found in the extracellular milieu of WT cohort. These data corroborate the ex vivo retinal Abeta immunohistochemistry, that showed no RGCs labeled and only a few extracellular Abeta immunoreactive spots detected in WT mice (Figure 2A, red, 546 nm channel).

Quantitative analysis of the ThioS fluorescence was measured globally by combining treated 5XFAD and WT data for thresholding the information using Otsu’s method [23]. This threshold was applied to each dataset to create binary images representing dark and bright pixels; the overall numbers of bright pixels were counted. As shown in Figure 2B, the number of Abeta deposits in 5XFAD retina is approximately four-fold higher compared to WT mice (*p* < 0.05).

#### 2.2.2. Cross-Section Analysis

**Figure 3:**
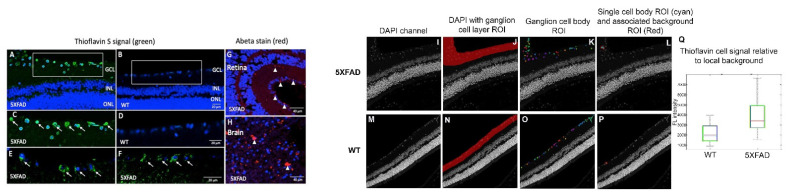
Imaging retinal Abeta using inhalable ThioS and corroboration with Abeta immunohistochemistry in the retina and brain. Fluorescence imaging of retinal cross-sections of 5XFAD (**A**) and WT controls (**B**) after ThioS inhalation. Higher magnifications of boxed areas in (**A**,**B**) are shown in (**C**,**D**). Arrows point to ThioS-labeled RGCs. Additional examples are shown in (**E**,**F**) of 5XFAD retina after ThioS inhalation. The retina (**G**) and brain (cerebral cortex) (**H**) of a 5XFAD mouse after immunohistochemical detection of Abeta. Red immunoreactivity is seen in small deposits in GCL and inner plexiform layer (IPL) of the retina and in plaques in the brain (arrowheads). Green (488 nm) channel demonstrates ThioS fluorescence; red (546 nm) channel depicts Abeta immunohistochemsitry; blue (405 nm) depicts DAPI labeling for nuclei. GCL = ganglion cell layer. INL = inner nuclear layer. ONL = outer nuclear layer. On the DAPI image (**I**,**M**), the ganglion cell layer was segmented manually (**J**,**N**). Then, an Otsu’s method threshold followed by a dilation/erosion step was used to identify cell body ROIs (**K**,**O**). The non-cell body pixel within 20 pixels of each cell body ROI was used for a measure of local background intensity (example for a single ROI in **L**,**P**). Then, applying the ROIs to the ThioS images, the mean intensity of local background pixels was subtracted from the mean intensity of each cell body ROI. The results for all cells are summarized in the boxplot for a 5XFAD (**I**–**L**) and a WT (**M**–**P**) mouse. Values for all cell body ROIs (**Q**).

In a different cohort of animals, WT (*n* = 2) and 5XFAD (*n* = 3) mice were treated with inhalable ThioS, then the cross-sections of the mouse retinae were prepared in which retinal layers were visualized with DAPI (blue) nuclear labeling of DNA. It is apparent that there is a remarkable distribution of ThioS in the retina. Particularly, the fluorescent signal of ThioS was found in the ganglion cell layer (GCL) (Figure 3A,C), associated with the cytoplasm of the ganglion cells of 5XFAD mice (Figure 3E,F). Meanwhile, minimal labeling is detected in the treated WT, aged, and sex-matched counterparts (Figure 3B,D). To corroborate the retinal imaging data of ThioS with the expression of Abeta in the retina, immunohistochemistry (IHC) with an antibody against Abeta was performed. In congruence with the imaging data, IHC showed abundant overexpression of Abeta deposits in the inner plexiform layer (IPL) and the cytoplasm of the ganglion cells (Figure 3G).

### 2.3. Abeta Deposits in the Retina Corroborates with Abeta Expression in the Brain of 5XFAD Mice

Next, it is necessary to confirm the tested 5XFAD mice also have the overexpression of Abeta in the cerebral cortical areas, and how that relates to Abeta deposits in the eyes within the individual subject. After perfusion, aside from collecting the ocular tissues, the brain was also removed and processed. As seen in Figure 3H, the brains of these corresponding mice also have abundant levels of Abeta. It is apparent that the expression of Abeta in the brain is more affluent and aggregated with large sizes compared to those found in the retina.

### 2.4. Data Segmentation Confirms the Distribution of ThioS within Ganglion Cell Layer

To compare the fluorescence intensity semi-quantitatively, ThioS image pixel values were measured in ganglion cells (Figure 3K,O) relative to the adjacent background (Figure 3L,P). Values for all cell body ROIs were summarized in the box-whisker graph in Figure 3Q, indicating that the probe indeed accumulates in the 5XFAD ganglion cells and the cell body pixel intensity of ThioS was greater in ganglion cells for the 5XFAD mouse compared to WT after background correction.

### 2.5. Double Staining Data on Retinal Cross-Sections Confirm ThioS Binds Abeta

**Figure 4:**
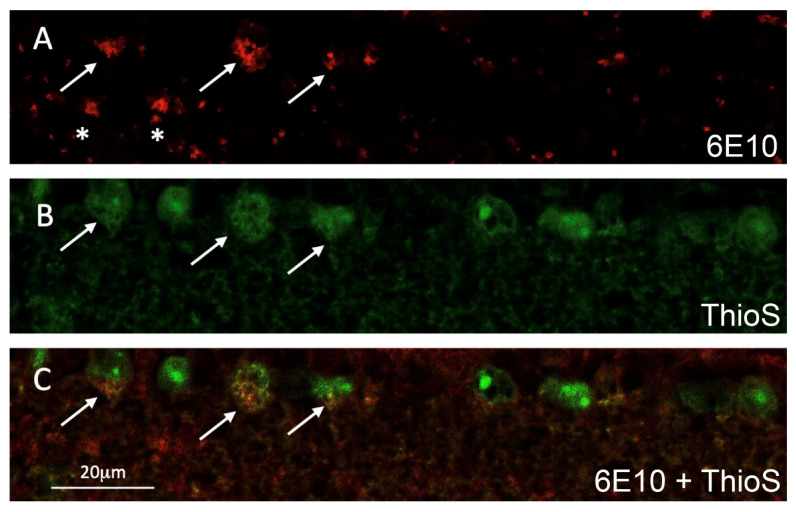
Representative confocal fluorescence imaging of a double-labeled retinal cross-section of a 5XFAD mouse after ThioS inhalation; (**A**) Abeta immunoreactivity (red, 546 nm channel) in retinal ganglion cells (RGC) shown by arrows and extracellular Abeta deposits shown by asterisks; (**B**) ThioS is seen in several RGCs (green, 488nm channel). Arrows indicate those ThioS labeled RGCs also immunolabeled for Abeta; (**C**) merged images of A and B showing double-labeled RGCs (arrows, orange).

Consecutive slides of the retinal cross-sectional tissues from ThioS-treated 5XFAD (shown in Figure 3) were subjected to additional immunohistochemistry staining against Abeta using 6E10 antibodies. The data demonstrated that green fluorescence label of ThioS is within the cytoplasmic compartment of RGC and corroborates the immunolabeling for Abeta shown in the red fluorescence in some of the RGCs (Figure 4). We noted that the fluorescence signal depicted from ThioS seems to be less sensitive compared to immunofluorescence data (Figure 4A,B). It is likely due to the low quantum efficiency of ThioS. Future study using Abeta-binding molecules with high quantum yield would alleviate this issue.

## 3. Discussion

We have developed a combination of ThioS formulation and a semi-high throughput nebulizer system for in vivo treatment of 5XFAD mice via aerosolization for the detection of retinal Abeta. The expression of Abeta deposits in this transgenic mouse model is found mostly in the retinal ganglion cell layer.

The data show that ThioS formulation is stable, and it can be conveniently stored at room temperature without any sign of degradation. Most notably, the concentration of the formulated solution was calibrated to ensure appropriate contrast capability while rendering the ThioS molecule in water during the formulation process, a condition ideal for preclinical and clinical operations. As the nebulization output is a function of fluid viscosity and surface tension, reducing one of these factors, for instance, in this case, using water instead of a viscous solution, would increase aerosol output and reduce nebulization time [24].

The involvement of retinal tissue in AD found in this work is congruent with the fact that the eye functions as a window to the brain from where the retinal processing of visual input is transferred to the higher brain centers via the retinal ganglion cells, the major output cell of the retina [25]. As a part of the central nervous system (CNS), the retina shares common features with the brain, including embryological development, anatomy, and function [26,27,28]. Axons from the optic nerve form a direct connection between the retina and the brain and facilitate the transport of amyloid precursor protein (APP) created in retinal ganglion cells [29]. There is increasing evidence that the retinal ganglion cell axons in the nerve fiber layer as well as the inner retinal layers are affected in early AD pathology [30,31]. Further, clinical data also corroborate a connection between AD and visual impairment as visual dysfunction is not only common in AD patients, but it is also evident in early stages of AD [32,33,34]. In the past, our group also showed that Abeta deposits in the inner retina, resulted in retinal degeneration in a mouse model of AD [18]. Recently, retinal Abeta imaging for AD diagnosis has been demonstrated in a proof-of-concept clinical trial [20].

Our data showed that the inhalable ThioS can detect Abeta deposits in the retinae of animal models. The fluorescent signal depicts morphologically distinct intracellular and extracellular Abeta deposition, albeit significantly higher in 5XFAD mice compared to WT counterparts. Interestingly, we observed extracellular Abeta deposition in several regions of the 5XFAD mouse retina. While significant intracellular load of Abeta in the RGCs of 5XFAD mice was detected, but no such indication found in WT animals. This observation of intracellular versus extracellular Abeta deposition in the retinae resonates with the current debate about the role of intracellular Abeta in AD pathogenesis [35]. More work is necessary to address this question. Yet, for Abeta in the brain, the implications of intraneuronal Abeta in AD provide new insight into the link between Abeta and tau pathologies that were initially considered as two separate pathologies [36].

Detection of Abeta deposits in the retina benefits AD research and clinical diagnosis in many ways. First, the availability of retinal imaging devices offers non-invasive in vivo imaging at sub-cellular resolutions [37,38]. Novel retinal imaging devices are compact, economical, and can be set up in ophthalmology and neurology clinics across the country. Further, the compatibility of preclinical and clinical devices facilitates the translation of data from animals to human. Second, this approach uses rather safe and ready-to-use fluorescent reporter probes unlike the invasive administration of radioisotopes in the case of PET imaging for Abeta targets in the brain, making populational screening for AD a reality in the near future. In the past, a curcumin derivative has been used as a promising probe to visualize Abeta deposits in the retina for both preclinical and clinical trials [20,39]. Third, one of the daunting tasks in brain imaging involves the blood-brain barrier (BBB), which excludes the majority of the probes from entering the brain parenchyma. Imaging in the retina encounters the blood-retinal barrier (BRB), which comprises the endothelial cells of retinal blood vessels of the inner BRB and the retinal pigment epithelial cells of the outer BRB. Different from the BBB, retinal vessels have a higher density of interendothelial junctions and endothelial vesicles, resulting in higher vascular permeability [40]. Further uptake studies confirmed this observation, proving that the retinal uptake indexes of several bioactive materials were approximately fourfold higher than the brain uptake index values [41]. Overall, retinal imaging will help to repurpose and realize the potentials of a number of Abeta reporters that otherwise have no application in vivo due to their inability to traverse across the BBB. This approach eliminates expenses and difficulties in setting up assays to screen for new Abeta reporters for the brain, and many other problems encountered that make the development of new probes for the brain an extremely long and complicated process. The use of aerosolized ThioS for imaging retinal Abeta reported here exemplifies this very notion.

## 4. Materials and Methods

### 4.1. Animals

A colony of 5XFAD mice obtained from Jackson Laboratories was maintained by crossing with WT BL/6J as we reported in the past [16]. The mice were genotyped by polymerase chain reaction (PCR) of a tail or ear DNA with the following primers: PSEN1 forward, 5′-TCATGACTATCCTCCTGGTGG-3′, and reverse, 5′-CGTTATAGGTTTTAAACACTTCCCC-3′. For APP, forward, 5′ -AGGACTGACCACTCGACCAG-3′ and reverse, 5′-CGGGGGTCTAGTTCTGCAT-3′. After PCR amplification, DNA product was analyzed using a 1% agarose gel; APP transgene = 377 bp, and PSEN1 transgene = 608 bp. 5XFAD mice were maintained as heterozygous. Animal experiments were conducted per the guidelines established by the Vanderbilt University’s Institutional Animal Care and Use Committee (IACUC) and the Division of Animal Care. The performed work was approved by Vanderbilt IACUC with an extended protocol, M1700044-01.

### 4.2. ThioS Formulation and Characterization

The overall objective is to demonstrate the proof-of-principle to generate candidate formulation to advance in vivo application of aerosolized ThioS. First, 2% ThioS was prepared in dd water (*w*/*v*) at room temperature with moderate stirring using a magnet stirrer. The resulted solution was filtered to eliminate any residual occupying materials in the mixture using 45 μm Luer-Lok syringe filters. ThioS aerosol quality was determined by collecting the entrapped aerosol after passing through the atomizer using a cartridge fitted with a pre-weighed filter paper. The ThioS was extracted from the filter paper using acetonitrile and water (50:50), and the solution was removed using a rotavapor. Then, the residual was completely removed using a lyophilizer. The integrity of ThioS was characterized using a spectrophotometer comparing to the mother solution. The whole process was performed in the dark or the materials were wrapped with aluminum foil to prevent photobleaching.

### 4.3. Preclinical Treatment of Mice with Aerosolized ThioS

The nebulizer (Figure 1A) was calibrated, and airflow was assessed as a function of pressure to ensure a consistent airflow rate (3 L/min) and an operating pressure of approximately 30 psi. The ThioS solution was delivered to the atomizer via a high-pressure syringe pump at a rate of 50 mL/h. During treatment, awake mice (12–16-month-old) (control, *n* = 4 vs. 5XFAD, *n* = 6) were placed inside a conical tube that was inserted into the exposure chamber. The animal’s snout was secured within the restraint tube by a stainless-steel nose cone. The nose cone is designed in a way that ensures animals inhale only freshly generated aerosol, while the exhaled materials are conveyed through a different channel that leads to the exhaust. Approximately 3h of treatment was required to administer an approximate dose of 5 mg/kg. About 1 h after the nebulization, animals underwent cardiac perfusion, followed by tissue extraction for analysis.

### 4.4. Cardiac Perfusion Procedure and Tissue Collection

After aerosol treatment, heavily anesthetized animals were laid on ice and the thoracic cavity was accessed using a scalpel, and the incision was stabilized with a retractor. Perfusion was performed as we described before [42], by slowly injecting ice-cold PBS (30 mL, pH 7.4) in the left ventricle using a 25-gauge syringe while the right atrium was snipped off to facilitate drainage of the systemic venous return. Then, 30 mL of 4% paraformaldehyde (PFA) (pH 7.4) was perfused. When completed, bilateral eye enucleation was performed. Forceps were used to press on the orbit until the eyeball protruded. The forceps were placed behind the eye, gripping the optical nerves tightly while circular movements of the forceps were made until the eyeball was detached. The animal was decapitated, and the brain was harvested and fixed in 4% PFA overnight at 4 °C. Brains and some eyes were cryoprotected for two days in 10% sucrose at 4 °C, and embedded in Tissue-Tek optimum cutting temperature (OCT) compound for cryosectioning, stored at −80 °C. All other fixed tissues were embedded in paraffin or processed as free-floating whole-mount retina.

### 4.5. Endogenous ThioS Fluorescence Imaging on Ex Vivo Retinal Whole-Mount Tissues

The whole-mount retina was prepared using a modification of the reported method [43]. Briefly, the cornea was removed by cutting in a circular path along the ora serrata with small scissors, holding the eye at the limbus with forceps. Next, the lens and vitreous were removed with blunt-edged forceps. The retina was then carefully detached from the eyecup by positioning forceps between the retina and the eyecup moving the forceps slowly around the circumference. The optic nerve was cut at the location between the retina and the eyecup to ease the separation of the retina from the eyecup. Once the whole-mount was separated, it was rinsed with PBS and strands of vitreous were removed under a dissection microscope. The whole-mount was washed in PBS (3X, pH7.4), 2–3 relieving cuts were made to allow the eyecup to flatten upon placement on a glass side. Next, whole-mount tissue was covered with aqueous mounting medium and coverslipped. Confocal images were taken at excitation wavelength of 488 nm for ThioS (Figure 2A top) using a Zeiss 800 confocal microscope (Carl Zeiss, White Plains, NY, USA).

### 4.6. Ex Vivo Whole-Mount and Abeta Immunofluorescence

Some of the whole-mounts were processed for Abeta immunofluorescence (Figure 2A bottom). Free-floating whole-mounts were washed in PBS (3X, pH7.4) then underwent antigen retrieval in 88% formic acid for 5 min at room temperature. The whole-mounts were washed again in PBS (3X, pH7.4) and then incubated in 3% normal goat serum in 0.3% TX-100 PBS for 20 min at room temperature. The whole-mount was then incubated in 6E10 mouse monoclonal antibody to Abeta (Covance, Princeton, NJ, USA), diluted in normal goat serum and PBS with 0.3% TX-100 at a working concentration of 1:100 for 1 h at RT and then overnight at 4 °C. Next, the whole-mounts were washed in PBS, then incubated with a 1:400 secondary antibody goat anti-mouse Cy3 Alexa 546 (Thermofisher Scientific, Grand Island, NY, USA) in PBS pH 7.4 for 45 min at room temperature. The whole-mounts were washed again and then incubated for nuclear staining in 1:500 DAPI for 5 min at room temperature, then rinsed for 1h in PBS. Next, the whole-mounts were placed on glass slides and covered with aqueous mounting medium for confocal imaging. Confocal images were taken at excitation wavelength of 546 nm for Cy3 and 405 nm for DAPI using a Zeiss 800 confocal microscope (Carl Zeiss, White Plains, NY, USA).

### 4.7. Ex Vivo Brain and Retinal Cross-Sections Processed for Abeta Immunofluorescence and ThioS Fluorescence

Frozen cross-sections of eye and brain were air dried and rinsed in PBS (3X, pH7.4). Cross-sections underwent antigen retrieval in 88% formic acid for 5 min at room temperature and then rinsed in PBS. Next, blocking was done with 3% normal goat serum in 0.3% TX-100 PBS for 20 min at room temperature. The sections were then incubated in BA4 monoclonal mouse anti-human beta-amyloid clone 6F/3D (Agilent, Santa Clara, CA, USA) primary antibody using 1:100 concentration in 3% normal goat serum and 0.3% TX100 PBS at room temperature for 1 h then at 4 °C overnight. Negative control slides were prepared by incubation with Mouse IgG1 isotype. Sections were then rinsed in PBS and incubated in goat anti-mouse Alexa 546 secondary antibody (Thermofisher Scientific, Grand Island, NY, USA) at 1:400 in PBS pH 7.4 for 45 min at room temperature. The sections were then rinsed again and incubated for nuclear staining in 1:500 DAPI for 5 min at room temperature and then rinsed for 1 h in PBS. Finally, the sections were coverslipped with aqueous mounting medium and imaged for Cy3 546 nm for Abeta immunofluorescence and 405 nm for DAPI labeled nuclei (Figure 3G,H).

Paraffin cross-sections were also used for Abeta immunohistochemistry. Primary antibody 6E10 mouse monoclonal antibody to Abeta (Covance, Princeton, NJ, USA) at 1:100 concentration was used instead of BA4 antibody above. Staining procedures were the same as BA4 primary antibody mentioned above except the primary antibody was replaced by 6E10 antibody. Negative control slides were incubated with Mouse IgG1 isotype. The sections were rinsed again and incubated for nuclear staining in 1:500 DAPI for 10 min at room temperature and then rinsed for 1 h in PBS. The 6E10 Abeta immunoreactivity was also imaged with confocal microscopy with Cy3 Alexa 546 nm channel (Figure 4A).

Some paraffin cross-sections from treated 5XFAD animals were also used for ThioS imaging. Sections underwent Abeta immunohistochemistry as described above using the 6E10 antibody, and then coverslipped for confocal microscopy. Next, confocal imaging was performed using 488 nm channel for endogenous ThioS fluorescence and the 546 nm channel for Abeta immunofluorescence (Figure 4B,C).

### 4.8. Signal Thresholding for Semi-Quantitative Data Analysis

The ganglion cell layer was delineated manually on the DAPI image (Figure 3J,N). In the ganglion cell ROI, a threshold between background and cell body signal was determined using Otsu’s method. Connected regions of pixels with intensity greater than 2x the threshold, and of minimum area 500 pixels were identified. These regions were dilated, then eroded, with a disk kernel of 5-pixel radius to produce a series of cell body ROIs (Figure 3K,O). To obtain a background ROI for each cell body ROI, the cell body ROI was dilated with a disk kernel of 20-pixel radius, and all non-cell-body pixels in the dilated ROI that were not included in any cell body were retained for the local background ROI (example, Figure 3L,P showing one cell body ROI and its associated background ROI). The mean signal from each cell body ROI was extracted from the green-channel image, and the mean signals from the adjacent background ROI was subtracted to produce the background-adjusted pixel intensity measurement for the particular cell body ROI.

### 4.9. Statistical Analysis

The experimental data were reported with standard Wilcoxon matched-pairs two-tailed rank test between cohorts of mice using GraphPad software (GraphPad Prism, San Diego, CA, USA). *P* values are two-tailed and differences with *p* values < 0.05 were considered statistically significant.

## 5. Conclusions

In summary, the presented methodology will open up a new avenue for in vivo application of ThioS and many related Abeta-binding compounds, such as Congo red, thioflavin T or targeted probes, capable of the detection of soluble Abeta oligomers to improve early AD diagnosis [44]. Although Abeta in the brain is also detected by inhalable Abeta-binding molecules [22], the ability to undertake in vivo optical brain imaging is challenging since the brain is surrounded by the skull and areas of the brain that accumulate Abeta are often deep structures. However, the detection of a specific Abeta-binding probe in the retina is clinically relevant, and feasible due to the clear, transparent ocular structures (e.g., cornea and lens) of the visual axis. If successful, imaging Abeta deposits in the retina has the potential to serve as a surrogate method to assess Abeta plaques in the brain, which can not only facilitate early detection of the disease but could also be used to monitor Abeta plaque reduction in response to drug therapy [45].

Aside from all of the advantages discussed above, there remain a few important caveats that need to be addressed to move this field forward. As retinal Abeta imaging has started to pick up more momentum, it is worth cross validating the information with other imaging modalities, along with using a standard format, to reliably compare data across institutions, as mentioned in a recent review article [28]. Future work should be performed using different mouse models in order to address the possible differences in Abeta temporal expression between animals. Moreover, bringing new data analysis expertise, as demonstrated in this work, creating new segmentation algorithms and data processing is crucial to standardize data analysis, reducing discrepant results obtained from one lab to another. Last but not least, the correlation of retinal Abeta to those in the brain using other biomarkers, such as amyloid PET, and staging the disease with a longitudinal assessment of cognitive functions is in our agenda.

## Data Availability

Data available on request from the corresponding authors.

## Sample Availability

Samples of the compounds are not available from the authors.

## References

[1] Selkoe D.J. (2011). Alzheimer’s disease. Cold Spring Harb. Perspect. Biol..

[2] Klunk W.E., Engler H., Nordberg A., Wang Y., Blomqvist G., Holt D.P., Bergstrom M., Savitcheva I., Huang G.F., Estrada S. (2004). Imaging brain amyloid in Alzheimer’s disease with Pittsburgh Compound-B. Ann. Neurol..

[3] Omachi Y., Ito K., Arima K., Matsuda H., Nakata Y., Sakata M., Sato N., Nakagome K., Motohashi N. (2015). Clinical impact of C-Pittsburgh compound-B positron emission tomography carried out in addition to magnetic resonance imaging and single-photon emission computed tomography on the diagnosis of Alzheimer’s disease in patients with dementia and mild cognitive impairment. Psychiatry Clin. Neurosci..

[4] Liu E., Schmidt M.E., Margolin R., Sperling R., Koeppe R., Mason N.S., Klunk W.E., Mathis C.A., Salloway S., Fox N.C. (2015). Amyloid-beta 11C-PiB-PET imaging results from 2 randomized bapineuzumab phase 3 AD trials. Neurology.

[5] (2013). GE beta-amyloid agent approved. J. Nucl. Med..

[6] Yang L., Rieves D., Ganley C. (2012). Brain amyloid imaging—FDA approval of florbetapir F18 injection. N. Engl. J. Med..

[7] Kung M.P., Hou C., Zhuang Z.P., Skovronsky D.M., Zhang B., Gur T.L., Trojanowski J.Q., Lee V.M., Kung H.F. (2002). Radioiodinated styrylbenzene derivatives as potential SPECT imaging agents for amyloid plaque detection in Alzheimer’s disease. J. Mol. Neurosci..

[8] Swan A., Waddell B., Holloway G., Bak T., Colville S., Khan Z., Pal S. (2015). The diagnostic utility of 99mTc-HMPAO SPECT imaging: A retrospective case series from a tertiary referral early-onset cognitive disorders clinic. Dement. Geriatr. Cognit. Disord..

[9] Zhen W., Han H., Anguiano M., Lemere C.A., Cho C.G., Lansbury P.T. (1999). Synthesis and amyloid binding properties of rhenium complexes: Preliminary progress toward a reagent for SPECT imaging of Alzheimer’s disease brain. J. Med. Chem..

[10] Zhuang Z.P., Kung M.P., Hou C., Skovronsky D.M., Gur T.L., Plossl K., Trojanowski J.Q., Lee V.M., Kung H.F. (2001). Radioiodinated styrylbenzenes and thioflavins as probes for amyloid aggregates. J. Med. Chem..

[11] Higuchi M., Iwata N., Matsuba Y., Sato K., Sasamoto K., Saido T.C. (2005). 19F and 1H MRI detection of amyloid beta plaques in vivo. Nat. Neurosci..

[12] Yanagisawa D., Taguchi H., Ibrahim N.F., Morikawa S., Shiino A., Inubushi T., Hirao K., Shirai N., Sogabe T., Tooyama I. (2014). Preferred features of a fluorine-19 MRI probe for amyloid detection in the brain. J. Alzheimer’s Dis..

[13] Aoki I., Wu Y.J., Silva A.C., Lynch R.M., Koretsky A.P. (2004). In vivo detection of neuroarchitecture in the rodent brain using manganese-enhanced MRI. NeuroImage.

[14] Bade A.N., Gendelman H.E., Boska M.D., Liu Y. (2017). MEMRI is a biomarker defining nicotine-specific neuronal responses in subregions of the rodent brain. Am. J. Transl. Res..

[15] Fontaine S.N., Ingram A., Cloyd R.A., Meier S.E., Miller E., Lyons D., Nation G.K., Mechas E., Weiss B., Lanzillotta C. (2017). Identification of changes in neuronal function as a consequence of aging and tauopathic neurodegeneration using a novel and sensitive magnetic resonance imaging approach. Neurobiol. Aging.

[16] Barton S.M., Janve V.A., McClure R., Anderson A., Matsubara J.A., Gore J.C., Pham W. (2019). Lipopolysaccharide induced opening of the blood brain barrier on aging 5XFAD mouse model. J. Alzheimer’s Disease.

[17] Hart N.J., Koronyo Y., Black K.L., Koronyo-Hamaoui M. (2016). Ocular indicators of Alzheimer’s: Exploring disease in the retina. Acta Neuropathol..

[18] Ning A., Cui J., To E., Ashe K.H., Matsubara J.A. (2008). Amyloid-beta deposits lead to retinal degeneration in a mouse model of Alzheimer disease. Investig. Ophthalmol. Vis. Sci..

[19] Jorge L., Canario N., Martins R., Santiago B., Santana I., Quental H., Ambrosio F., Bernardes R., Castelo-Branco M. (2020). The Retinal Inner Plexiform Synaptic Layer Mirrors Grey Matter Thickness of Primary Visual Cortex with Increased Amyloid beta Load in Early Alzheimer’s Disease. Neural Plast..

[20] Koronyo Y., Biggs D., Barron E., Boyer D.S., Pearlman J.A., Au W.J., Kile S.J., Blanco A., Fuchs D.T., Ashfaq A. (2017). Retinal amyloid pathology and proof-of-concept imaging trial in Alzheimer’s disease. JCI Insight.

[21] McClure R., Ong H., Janve V., Barton S., Zhu M., Li B., Dawes M., Jerome W.G., Anderson A., Massion P. (2017). Aerosol Delivery of Curcumin Reduced Amyloid-beta Deposition and Improved Cognitive Performance in a Transgenic Model of Alzheimer’s Disease. J. Alzheimer’s Dis..

[22] McClure R., Yanagisawa D., Stec D., Abdollahian D., Koktysh D., Xhillari D., Jaeger R., Stanwood G., Chekmenev E., Tooyama I. (2015). Inhalable curcumin: Offering the potential for translation to imaging and treatment of Alzheimer’s disease. J. Alzheimers Dis..

[23] Otsu N. (1979). A Threshold selection method from gray-level histogram. IEEE Trans. Syst. Man Cybern..

[24] McCallion O.N., Taylor K.M., Thomas M., Taylor A.J. (1995). Nebulization of fluids of different physicochemical properties with air-jet and ultrasonic nebulizers. Pharm. Res..

[25] Roth S. (2015). Inhaled anesthesia, apoptosis, and the developing retina: A window into the brain?. Anesth. Analg..

[26] Byerly M.S., Blackshaw S. (2009). Vertebrate retina and hypothalamus development. Wiley Interdiscip. Rev. Syst. Biol. Med..

[27] Maude R.J., Dondorp A.M., Abu Sayeed A., Day N.P., White N.J., Beare N.A. (2009). The eye in cerebral malaria: What can it teach us?. Trans. R. Soc. Trop. Med. Hyg..

[28] Trost A., Lange S., Schroedl F., Bruckner D., Motloch K.A., Bogner B., Kaser-Eichberger A., Strohmaier C., Runge C., Aigner L. (2016). Brain and Retinal Pericytes: Origin, Function and Role. Front. Cell Neurosci..

[29] Alber J., Goldfarb D., Thompson L.I., Arthur E., Hernandez K., Cheng D., DeBuc D.C., Cordeiro F., Provetti-Cunha L., den Haan J. (2020). Developing retinal biomarkers for the earliest stages of Alzheimer’s disease: What we know, what we don’t, and how to move forward. Alzheimer’s Dement..

[30] Lu Y., LI Z., Zhang X., Ming B., Jia J., Wange R., Ma D. (2010). Retinal nerve fiber layer structure abnormailities in early Alzheimer’s disease. Neurosci. Lett..

[31] Moschos M.M., Markopoulos I., Chatziralli I., Rouvas A., Papageorgiou S.G., Ladas I., Vassilopoulos D. (2012). Structural and functional impairment of the retina and optic nerve in Alzheimer’s disease. Curr. Alzheimer Res..

[32] Gilmore G.C., Wenk H.E., Naylor L.A., Koss E. (1994). Motion perception and Alzheimer’s disease. J. Gerontol..

[33] Javaid F.Z., Brenton J., Guo L., Cordeiro M.F. (2016). Visual and Ocular Manifestations of Alzheimer’s Disease and Their Use as Biomarkers for Diagnosis and Progression. Front. Neurol..

[34] Yamasaki T., Horie S., Ohyagi Y., Tanaka E., Nakamura N., Goto Y., Kanba S., Kira J., Tobimatsu S. (2016). A Potential VEP Biomarker for Mild Cognitive Impairment: Evidence from Selective Visual Deficit of Higher-Level Dorsal Pathway. J. Alzheimer’s Dis..

[35] Gouras G.K., Tampellini D., Takahashi R.H., Capetillo-Zarate E. (2010). Intraneuronal beta-amyloid accumulation and synapse pathology in Alzheimer’s disease. Acta Neuropathol..

[36] Takahashi R.H., Capetillo-Zarate E., Lin M.T., Milner T.A., Gouras G.K. (2010). Co-occurrence of Alzheimer’s disease ss-amyloid and tau pathologies at synapses. Neurobiol. Aging.

[37] Lim J.K., Li Q.X., He Z., Vingrys A.J., Wong V.H., Currier N., Mullen J., Bui B.V., Nguyen C.T. (2016). The Eye As a Biomarker for Alzheimer’s Disease. Front. Neurosci..

[38] London A., Benhar I., Schwartz M. (2013). The retina as a window to the brain-from eye research to CNS disorders. Nat. Rev. Neurol..

[39] Koronyo Y., Salumbides B.C., Black K.L., Koronyo-Hamaoui M. (2012). Alzheimer’s disease in the retina: Imaging retinal abeta plaques for early diagnosis and therapy assessment. Neurodegener. Dis..

[40] Stewart P.A., Tuor U.I. (1994). Blood-eye barriers in the rat: Correlation of ultrastructure with function. J. Comp. Neurol..

[41] Toda R., Kawazu K., Oyabu M., Miyazaki T., Kiuchi Y. (2011). Comparison of drug permeabilities across the blood-retinal barrier, blood-aqueous humor barrier, and blood-brain barrier. J. Pharm. Sci..

[42] McClure R.A., Chumbley C.W., Reyzer M.L., Wilson K., Caprioli R.M., Gore J.C., Pham W. (2013). Identification of promethazine as an amyloid-binding molecule using a fluorescence high-throughput assay and MALDI imaging mass spectrometry. NeuroImage Clin..

[43] Ivanova E., Toychiev A.H., Yee C.W., Sagdullaev B.T. (2013). Optimized protocol for retinal wholemount preparation for imaging and immunohistochemistry. J. Vis. Exp..

[44] Tonali N., Dodero V.I., Kaffy J., Hericks L., Ongeri S., Sewald N. (2020). Real-Time BODIPY-Binding Assay To Screen Inhibitors of the Early Oligomerization Process of Abeta1-42 Peptide. ChemBioChem.

[45] Sidiqi A., Wahl D., Lee S., Ma D., To E., Cui J., To E., Beg M.F., Sarunic M., Matsubara J.A. (2020). In vivo Retinal Fluorescence Imaging With Curcumin in an Alzheimer Mouse Model. Front. Neurosci..

